# Variation in patient-reported outcomes after total hip replacement across ten high-volume hospitals in Germany: results from a multicenter, prospective, longitudinal Cohort Study

**DOI:** 10.1007/s10198-025-01858-4

**Published:** 2025-10-29

**Authors:** Martin Emmert, Cornelia Frömke, Cordula Drach, Laura Heppe, Susann Kiss, Christiane Patzelt, Anja Schindler, Oliver Schöffski, Jona Stahmeyer, Julia Weber, Uwe Sander

**Affiliations:** 1 Faculty of Law, Business & Economics, Institute for Healthcare Management & Health Sciences, University of Bayreuth, Prieserstraße 2, 95444 Bayreuth, Germany; 2 Bavarian Cancer Registry, Regional Registration Center Bayreuth, Meistersingerstraße 2, 95444 Bayreuth, Germany; 3 Department of Information and Communication, Faculty for Media, Information and Design, University of Applied Sciences and Arts, Hannover, Germany; 4 Chair of Health Care Management, School of Business and Economics, Friedrich-Alexander-University of Erlangen-Nuremberg, Lange Gasse 20, 90403 Nuremberg, Germany; 5 AOK Lower Saxony, Hannover, Germany

**Keywords:** Patient reported outcomes, Minimal important change, Mininmal important difference, Oxford Hip Score, Quality of care, Germany, I14

## Abstract

**Background:**

International studies have demonstrated hospital-related variations in the outcomes of total hip replacement (THR) based on clinical outcome measures and Patient-Reported Outcomes (PROs). Therefore, this study explored hospital-related variations for THR based on PROs in Germany.

**Methods:**

We performed a multicenter, prospective, longitudinal cohort study. Patients were recruited in ten high-volume hospitals in Lower Saxony, Germany (11/2019–2/2022). We measured the difference between the preoperative and 6-month postoperative absolute scores using the Oxford Hip Score (OHS). Therefore, we employed a multifactorial analysis of covariance model with OHS change from baseline as the dependent variable.

**Results:**

In total, 583 patients (65.54 ± 9.91 years; 62.1% female) were included in our analysis. The unadjusted mean OHS score increased from 21.61 ± 7.63 to 40.75 ± 8.10 points, thus indicating a change of 19.14 ± 9.58 points. Overall, 503 patients (86.3%) experienced a minimal important change (MIC) of at least 9 OHS points from the preoperative period to 6-months postoperative. The adjusted change in OHS points for participating hospitals varied between 13.41 and 17.99 OHS points. We found differences between the top-performing hospital and two hospitals (*p* < 0.05 each); however, those differences were shown to be below the minimal important difference (MID) of at least 5.22 OHS points.

**Conclusions:**

We identified differences in the quality of care for THR among high-volume hospitals in Germany; however, those differences were below the MID threshold. Furthermore, both higher preoperative OHS scores and lower rates of pain relief usage was associated with lower change scores. We recommend studies to explore hospital-related clinically relevant variation for THR which also include low-volume hospitals and an evaluation of both MIC and MID thresholds in Germany.

**Supplementary Information:**

The online version contains supplementary material available at 10.1007/s10198-025-01858-4.

## Introduction

Total hip replacement (THR) is performed annually in approximately one million patients worldwide [1]. In Germany, elective THR for patients with hip arthrosis or arthritis was performed 160,910 times in 1,254 hospitals in 2020 [2]. Although THR has shown to provide promising results in both reducing pain severity and improving the joint function of the patients, there is still potential for further improvement [1]. For example, hospital-related variations in the outcomes of THR could be shown to lead to longer hospital stays, an increased risk of postoperative complications, higher revision rates, and higher costs in low-performing hospitals [1]. In this context, Mufarrih and colleagues published a meta-analysis of 44 studies focusing on the impact of hospital volume on the outcomes of THR. As a result, it was shown that low-volume hospitals were associated with a higher rate of surgical site infections, 90-day complications, and mortality rates compared to high-volume hospitals [1]. In addition, another study from Germany determined significantly lower risk-adjusted in-hospital mortality rates for THR in high-volume hospitals compared to low-volume hospitals (0.10% vs. 0.23%) [3].

To date, studies assessing the volume-outcome relationship in elective surgery have focused on more traditional outcomes such as complications and postoperative mortality [4]. Regarding the latter, postoperative mortality might be of limited importance for quality assurance in THR since it occurs very rarely; for example, the 90-day mortality after THR in the United Kingdom was calculated to be 0.3% [4], while the in-hospital mortality rate following THR was 0.05% in Germany and the complication rate was 3.79% [2]. Despite this potentially limited importance for THA, both mortality and complication rates play an important role in German quality assurance systems. For example, all four hospital-related quality assurance measures of the quality assurance system of the German Statutory Health Insurance AOK (Allgemeine Ortskrankenkasse) (AOK-QSR) [5] and four of eight measures of the quality assurance system of the Institute for Quality Assurance and Transparency in Health Care (IQTIG) were related to mortality or complications [2].

So far, the minority of studies have measured the efficacy of THR – across hospitals – based on more patient-related outcomes, such as Patient-Reported Outcomes (PROs) [6, 7], which means with focus on “any aspect of a patient’s health status that comes directly from the patient” [8]. Basically, PROs address “feedback from patients about their health and functional status (mostly before and after surgery), measured on a quantified scale using generic or condition-specific instruments” [9]. More recently, increasing interest in collecting PROs in many industrialized countries (e.g., the United States, Germany, Sweden) could be observed [9–12]. PROs are increasingly seen as primary outcome measures in clinical trials, real-world studies, and arthroplasty registries (e.g., the National Patient Reported Outcome Measures (PROMs) program in England [4]) [13]. In addition, PROs are commonly used for research, audit, and clinical practice for patients undergoing THR [14, 15]. PROs have also been used to assess and compare the outcomes achieved by healthcare providers such as hospitals [16]; for example, the NHS in England provides PROs on a hospital-level basis for the public [17]. Additionally, PROs have been shown to be of major importance from the perspective of patients when choosing a hospital for THR surgery [18]. Nevertheless, the scarcity of studies using PROs as an outcome measure (health gain from surgery) is not surprising, since they are still not available as routine data in most healthcare systems [4].

Recently, public-reported data of the National Joint Registry in England from 2022 has demonstrated hospital-related variations for THR based on PROs. Both low-performing and top-performing hospitals could be identified by using the change in the Oxford Hip Score – as the most frequently used PROM in THR [19] – from the preoperative result to the 6-month postoperative score. Here, 20 Trusts (hospital units) (95% confidence interval) and 12 Trusts (99.8% confidence interval) out of the total of 278 Trusts had change-scores below the national average. On the other hand, 13 Trusts (95% confidence interval) and 6 Trusts (99.8% confidence interval) had change-scores above the national average [20]. A recently published study also demonstrated geographical variations in the outcomes of THR and also presented the characteristics of hospitals that were associated with these variations [21]. In another study, Varagunam and colleagues showed that high-volume surgeon outcomes were statistically significantly better than those from low-volume surgeons; for example, an additional 10 cases were associated with higher OHS scores (0.06) [4]. In addition, another study from England also found evidence of significant unexplained variation between hospitals using PROs after THR [22].

When detecting differences in the quality of care based on PROs, it is important to mention that differences – even if statistically significant – need to exceed a certain minimal limit to be regarded as clinically relevant [19]. Thereby, the minimal important change (MIC) is considered to detect a relevant change in PROs which is large enough to be appreciated from the patients´ perspective [15, 23, 24]. As mentioned by Deckey and colleagues, “their growing use in orthopedic research stems from the necessity to identify a metric, other than the *p*-value, to better assess the effect size of an outcome” [19]. In this context, the authors conducted a systematic review of calculated individual MIC values and their respective ranges, as well as an assessment of their applications in THA research. The authors showed that there were 45 unique PROMs for which 242 MICs were reported. As mentioned above, the OHS was the most frequently reported PROM. The review also showed that the median MIC value for the OHS was 9 points [19]. Based on this, patients need to achieve an increase of at least 9 OHS points from the preoperative period to 6-months postoperative to experience a clinically relevant improvement. Additionally, the between-group minimal important difference (MID), which allows the estimation of a clinically relevant difference in change scores when comparing two groups (e.g., which received surgery in two different hospitals) [15, 23, 24], was shown to be 5.22 points for the OHS [15].

### Research questions

Several international studies have assessed hospital-related variations in PROs following THR [4, 20–22]. However, the presented evidence has not assessed whether PRO-based hospital-related variations might also be observable in Germany. Therefore, the objective of this study is to address this research gap by answering the following two research questions: (1) Are there statistically significant differences in the OHS change-score for elective THR surgery in German high-volume hospitals? (2) Are there clinically relevant differences in the OHS change-score for elective THR surgery in German high-volume hospitals?

## Methods

### Study design

The present study was designed as a multicenter, prospective, longitudinal cohort study. It aimed to investigate whether the OHS reveals hospital-related variations in the PROs of THR in German high-volume hospitals. Therefore, we measured the outcome as the difference between the OHS scores six months after surgery compared to the OHS scores before surgery. We used the German version of the OHS with scores ranging from 0 to 48 with higher scores indicating better values (i.e., higher quality of life results). In this article, we refer to this endpoint as the OHS change from baseline (preoperative). Ethics approval for this study was obtained from the Friedrich-Alexander-University Erlangen-Nuremberg (Germany) Ethics Board (196_19 B).

Patients were recruited in ten high-volume or very high-volume hospitals for elective THR surgery (see Supplementary Material 1 for key characteristics of the participating hospitals) in the German Federal State Lower Saxony from November 2019 to February 2022. Patients who met the following broad inclusion criteria were included: (1) validated diagnosis of coxarthrosis (ICD-10: M16); (2) primary THR surgery in one of ten recruiting hospitals; (3) age 18 + years; (4) ability to fill out the questionnaire regarding physical, psychological, cognitive, or language aspects; and (5) informed consent. We then ranked all hospitals in Germany into quintiles of approximately equal case numbers according to their annual volume following the methodological approach used by Nimptsch and colleagues [3] to be able to compare the number of cases of the participating hospitals. Based on this, our study included four very high-volume hospitals and six high-volume hospitals. Furthermore, three hospitals were certified as an EndoProstheticsCenter, five as an EndoProstheticsCenter of Maximum Care and two hospitals could not be shown to be certified. Based on the results of the quality assurance system of the German Statutory Health Insurance AOK (see above; AOK-QSR) [5], one hospital was considered to be a top-performer, six hospitals to be middle-performer, and three hospitals were assigned to be low-performer. [5] Regarding the hospital ownership, seven were labeled as non-profit hospitals, two as public hospitals and one as a private hospital (see Supplemental Material 1 for an overview of key characteristics of participating hospitals.)

The preoperative OHS scores were obtained in a first step using a paper-based questionnaire before surgery in the corresponding hospitals; this questionnaire also included questions about self-reported existing health conditions. In the second step, patients were contacted via postal mail six months after surgery and received a printed version of the postoperative questionnaires; after two weeks, we sent out a reminder. The questionnaire was piloted by 20 patients to ensure the comprehensibility of the wording and internal validity; final adjustments were made accordingly. As an incentive for completing the questionnaires, respondents received a payment of 50€.

### Statistical methods

Besides conducting descriptive analysis, we developed an analysis of covariance (ANCOVA) model to assess the above-stated research questions. In this model, the OHS change from baseline (i.e., the difference between the mean preoperative and 6-month postoperative absolute OHS scores) served as the dependent variable. Regarding the selection of independent variables, we conducted the following steps: in the first step, we calculated univariable models; based on this, variables were selected for inclusion in the multivariable model if they were statistically significant at the 20% level using the global *F*-test. The strength of association between independent variables was tested in the second step using Cramérs V and cross tables. We also tested for second-order interactions between the selected variables and the variable representing the ten included hospitals. Interactions which could not be proven to be either statistically significant at the 20% level or medically justifiable were not considered to be relevant in the following analysis. The quantitative variables were tested for a linear, quadratic or cubic relationship to the OHS difference. In the next step, we developed a full model to adjust for the patient case mix including all variables with a significant univariable *p*-value of the global *F*-test (see above). Significant interactions with hospitals (main factors and interaction term) were also included, but removed from the model when the global *p*-value was larger than 20%. For associated variables, only one characteristic was included. Variables were considered to be significant if the associated global *p*-value from the ANCOVA model was below 5%. The same significance level (two-sided) was used for pairwise tests.

Since the present study should not be regarded as a confirmatory trial, a multiplicity correction to control the number of false positives in the statistical tests was not applied [25]. The clinical relevance of the results was assessed using estimators and two-sided 95% confidence intervals from the final model. As stated above, determining whether (statistically significant) changes in the OHS are also clinically meaningful is required for judging the efficacy of joint replacement. Therefore, one has to distinguish between changes in single groups of patients over time (i.e., cohort studies), for differences between groups and for assessing changes in individual patients [15]. In this context, the MIC is regarded to help clinicians and other stakeholders identify an improvement that is large enough to matter to an individual patient [15, 19, 23, 24]. As mentioned above, a recently published systematic review showed that the median MIC value for the OHS was 9 points [19]. Based on this, patients need to achieve an increase of at least 9 OHS points from the preoperative period to 6-months postoperative to experience a clinically relevant improvement. Additionally, the between-group minimal important difference (MID), which allows the estimation of a clinically relevant difference in change scores when comparing two groups (e.g., which received surgery in two different hospitals) [15, 23, 24], was shown to be 5.22 points for the OHS [15].

We also investigated further effects in subgroup analyses. These effects were analyzed using univariate tests for one or two samples. The tests were chosen depending on the distribution of the underlying data (e.g., t-test, Wilcoxon rank sum test). The significance level was set to 5% (two-sided). Statistical analyses were performed using SAS®, version 9.4 TS Level 1M7 (SAS Institute Inc., 2016).

## Results

### Sample characteristics

Overall, 583 patients were included in our analysis (Table 1) (see also Supplementary Material 2 for the patient flow chart). The mean age of patients was 65.54 (SD 9.91) years. Most respondents were female (62.1%) and stated secondary general school or lower as the highest educational level (42.5%). The mean preoperative OHS for all patients was 21.61 (SD 7.63). Overall, 382 patients (65.6%) rated their current health as good or better. Regarding treatment-related issues, most participants reported taking pain relief on a daily basis (40.3%). In addition, the most frequently reported pre-existing conditions were osteoarthritis (80.5%), back pain (56.3%) and high blood pressure (49.2%). Table 1 also presents the patient characteristics across all ten hospitals. As shown, the mean age varied between 62.57 (SD 9.49) years for hospital 3 and 68.17 (SD 11.56) years for hospital 10. The mean preoperative OHS score was lowest in hospital 3 (19.40; SD 6.04) and highest in hospital 8 (23.06; SD 7.60).

**Table 1:** Key characteristics for patients (*n* = 583)

	Overall sample (*n* = 583)		Hospitals									
	Mean (SD) or n	Range or %	1 (*n* = 55)	2 (*n* = 69)	3 (*n* = 21)	4 (*n* = 39)	5 (*n* = 70)	6 (*n* = 67)	7 (*n* = 65)	8 (*n* = 70)	9 (*n* = 69)	10 (*n* = 58)
Age												
Mean (SD)	65.54 (9.91)	25–86	65.56 (9.14)	65.30 (8.49)	62.57 (9.49)	65.00 (9.89)	62.61 (11.60)	66.78 (10.10)	67.65 (8.53)	66.40 (8.51)	63.62 (10.13)	68.17 (11.56)
Gender												
Male	221	37.9	26 (47.3%)	30 (43.5%)	6 (28.6%)	10 (25.6%)	22 (31.4%)	24 (35.8%)	33 (50.8%)	23 (32.9%)	25 (36.2%)	22 (37.9%)
Female	362	62.1	29 (52.7%)	39 (56.5%)	15 (71.4%)	29 (74.4%)	48 (68.6%)	43 (64.2%)	32 (49.2%)	47 (67.1%)	44 (63.8%)	36 (62.1%)
Preoperative OHS												
Mean (SD)	21.61 (7.63)	2–45	21.92 (7.31)	20.41 (7.04)	19.40 (6.04)	20.91 (8.14)	21.42 (7.39)	22.64 (7.86)	21.35 (7.51)	23.06 (7.60)	22.69 (8.31)	20.33 (7.98)
Preoperative Body-Mass-Index (BMI)												
Mean (SD)	28.78 (5.20)	16.02–54.29	29.62 (6.04)	29.55 (5.68)	30.44 (5.72)	29.08 (4.93)	28.41 (5.06)	29.21 (4.39)	27.64 (4.22)	29.04 (5.21)	28.19 (5.07)	27.91 (5.77)
Educational attainment												
Secondary general school or less	245	42.5	20 (37.0%)	26 (38.8%)	11 (55.0%)	16 (41.0%)	21 (30.4%)	40 (59.7%)	28 (43.1%)	25 (36.2%)	26 (37.7%)	32 (56.1%)
Intermediate secondary school	214	37.1	19 (35.2%)	32 (47.8%)	7 (35.0%)	17 (43.6%)	28 (40.6%)	17 (25.4%)	23 (35.4%)	25 (36.2%)	29 (42.0%)	17 (29.8%)
(Technical) University entrance qualification	118	20.5	15 (27.8%)	9 (13.4%)	2 (10.0%)	6 (15.4%)	21 (29.0%)	10 (14.9%)	14 (21.5%)	19 (27.5%)	14 (20.3%)	8 (14.0%)
Health Status												
Good or better	382	65.6	41 (74.5%)	42 (60.9%)	10 (47.6%)	25 (64.1%)	53 (75.7%)	42 (63.6%)	45 (69.2%)	47 (67.1%)	44 (63.8%)	33 (56.9%)
Satisfactory	166	28.5	11 (20.0%)	25 (36.2%)	6 (28.6%)	12 (30.8%)	12 (17.1%)	23 (34.8%)	16 (24.6%)	20 (28.6%)	19 (27.5%)	22 (37.9%)
Bad or worse	34	5.8	3 (5.5%)	2 (2.9%)	5 (23.8%)	2 (5.1%)	5 (7.1%)	1 (1.5%)	4 (6.2%)	3 (4.3%)	6 (8.7%)	3 (5.2%)
Year of diagnosis												
2 years and less ago	283	50.7	22 (41.5%)	41 (61.2%)	7 (35.0%)	24 (63.2%)	29 (43.9%)	33 (52.4%)	23 (38.3%)	34 (50.0%)	36 (54.5%)	34 (59.6%)
3–5 years ago	111	19.9	13 (24.5%)	9 (13.4%)	5 (25.0%)	4 (10.5%)	9 (13.6%)	12 (19.0%)	20 (33.3%)	22 (32.4%)	7 (10.6%)	10 (17.5%)
6–10 years ago	82	14.7	10 (18.9%)	11 (16.4%)	5 (25.0%)	5 (13.2%)	9 (13.6%)	9 (14.3%)	7 (11.7%)	5 (7.4%)	14 (21.2%)	7 (12.3%)
11 years ago or more	82	14.7	8 (15.1%)	6 (9.0%)	3 (15.0%)	5 (13.2%)	19 (28.8%)	9 (14.3%)	10 (16.7%)	7 (10.3%)	9 (13.6%)	6 (10.5%)
Intake of pain reliever												
Daily	234	40.3	22 (40.0%)	25 (37.3%)	8 (38.1%)	18 (46.2%)	30 (42.9%)	20 (29.9%)	30 (46.2%)	23 (32.9%)	26 (37.7%)	32 (55.2%)
4–6 times a week	62	10.7	2 (3.6%)	5 (7.5%)	4 (19.0%)	5 (12.8%)	11 (15.7%)	13 (19.4%)	4 (6.2%)	8 (11.4%)	7 (10.1%)	3 (5.2%)
2–3 times a week	97	16.7	8 (14.5%)	19 (28.4%)	3 (14.3%)	3 (7.7%)	12 (17.1%)	9(13.4%)	8 (12.3%)	13 (18.6%)	12 (17.4%)	10 (17.2%)
1 time a week	46	7.9	7 (12.7%)	5 (7.5%)	2 (9.5%)	2 (5.1%)	3 (4.3%)	10 (14.9%)	3 (4.6%)	9 (12.9%)	4 (5.8%)	1 (1.7%)
Less often or never	142	24.4	16 (29.1%)	13 (19.4%)	4 (19.0%)	11 (28.2%)	14 (20.0%)	15 (22.4%)	20 (30.8%)	17 (24.3%)	20 (29.0%)	12 (20.7%)
Pre-existing conditions												
Osteoarthritis	450	80.5	42 (79.2%)	47 (74.6%)	14 (73.7%)	32 (86.5%)	57 (82.6%)	49 (76.6%)	53 (84.1%)	56 (82.4%)	53 (79.1%)	47 (83.9%)
Back pain	315	56.3	32 (60.4%)	38 (62.3%)	11 (61.1%)	25 (65.8%)	38 (54.3%)	32 (50.0%)	35 (55.6%)	37 (54.4%)	30 (44.1%)	37 (64.9%)
High blood pressure	282	49.2	30 (55.6%)	36 (52.9%)	12 (60.0%)	21 (55.3%)	25 (35.7%)	40 (60.6%)	30 (46.2%)	33 (47.8%)	25 (37.3%)	30 (53.6%)
Gastrointestinal problems	67	12.1	5 (9.4%)	6 (9.7%)	4 (22.2%)	7 (18.4%)	6 (8.7%)	3 (4.5%)	8 (12.5%)	10 (14.7%)	9 (13.6%)	9 (17.3%)
Heart problems	66	11.8	4 (7.5%)	7 (11.3%)	3 (15.8%)	5 (12.8%)	6 (8.6%)	4 (6.0%)	8 (12.5%)	10 (14.7%)	10 (14.9%)	9 (18.0%)
Diabetes/Blood sugar	56	10.1	5 (9.4%)	11 (17.2%)	3 (16.7%)	5 (13.2%)	5 (7.1%)	10 (15.4%)	5 (7.9%)	2 (2.9%)	4 (6.1%)	6 (11.5%)
Lung problems	44	7.9	2 (3.8%)	7 (11.1%)	3 (15.8%)	3 (7.9%)	8 (11.6%)	3 (4.5%)	1 (1.6%)	5 (7.4%)	7 (10.6%)	5 (9.8%)
Depression	42	7.6	7 (13.2%)	5 (8.1%)	3 (17.6%)	2 (5.3%)	6 (8.6%)	4 (6.2%)	4 (6.3%)	3 (4.4%)	3 (4.5%)	5 (9.4%)
Rheumatism	35	6.3	0 (0.0%)	2 (3.2%)	2 (11.1%)	3 (7.9%)	4 (5.8%)	8 (12.5%)	4 (6.3%)	3 (4.5%)	4 (6.0%)	5 (9.8%)
Cancer	28	5.0	3 (5.7%)	6 (9.4%)	2 (10.5%)	2 (5.4%)	0 (0.0%)	2 (3.1%)	5 (7.8%)	2 (2.9%)	3 (4.5%)	3 (5.9%)

Please find in the following the number of missings [a detailed overview is presented in supplemental material 6]: Preoperative BMI (*n* = 2), Educational attainment (*n* = 6), Health Status (*n* = 1), Year of diagnosis (*n* = 25), Intake of pain reliever (*n* = 2), Osteoarthritis (*n* = 24), Back pain (*n* = 23), High blood pressure (*n* = 10), Gastrointestinal problems (*n* = 27), Heart problems (*n* = 24), Diabetes/Blood sugar (*n* = 26), Lung problems (n = 26), Depression (*n* = 28), Rheumatism (*n* = 30), Cancer (*n* = 28)

The preoperative and postoperative scores obtained using the 12-item OHS questionnaire are shown in Supplementary Material 3. The lowest preoperative and postoperative mean scores were reported for item 1 “Usual level of hip pain” (0.48 ± 0.64 and 2.86 ± 1.27, respectively). In contrast, the highest mean scores were calculated for item 2, “Trouble with washing and drying” (2.57 ± 0.93 and 3.61 ± 0.70, respectively). The lowest increase in OHS points from preoperative to 6-months postoperative was shown for item 2 “Trouble with washing and drying” (1.04 ± 1.04), item 5 “Doing household shopping alone” (1.05 ± 1.08) and item 6 “Walking time before severe pain” (1.05 ± 1.25). In contrast, the highest change in OHS points was shown for item 1 “Usual level of hip pain” (2.38 ± 1.36). It is important to mention that all changes in OHS scores from preoperative to 6-months postoperative were shown to be statistically significant (*p* < 0.001 each). Overall, the unadjusted mean OHS score increased from 21.61 ± 7.63 (preoperative) to 40.75 ± 8.10 (6-months postoperative) OHS points. Based on this, an overall improvement of 19.14 ± 9.58 OHS points could be determined.

Regarding the OHS scores on a hospital-based level, the mean preoperative OHS scores ranged between 19.40 ± 6.04 (hospital 3) and 23.06 ± 7.60 (hospital 8) (Table 2). The highest mean postoperative score was calculated to be 42.72 ± 6.05 (hospital 7); in contrast, the lowest postoperative score was reported to be 37.64 ± 10.04 (hospital 4). When comparing the improvement in OHS points from preoperative to 6-months postoperative, the highest difference could be observed for hospital 7 (21.38 ± 8.06), while the lowest difference was calculated for hospital 4 (16.72 ± 11.66). [Please also see Supplemental Material 4 presenting a ridgeline plot to display the distribution of the OHS difference by hospital.] Table 2 also shows the number and percentage of those patients who experienced a clinically relevant improvement (*n* = 503; 86.3%) and of those who did not (*n* = 80; 13.7%) on a hospital-based level; here, the increase in OHS points was below the MIC threshold of at least 9 OHS points. As shown (Table 2), the best results were calculated for hospital 7; here, the improvement in OHS points from preoperative to 6-months postoperative for 62 patients (95.4%) was shown to be clinically relevant. In contrast, the lowest results were calculated for hospital 4; here, only slightly more than three out of four patients (30/39; 76.9%) experienced a clinically relevant improvement.

**Table 2:** Preoperative and postoperative unadjusted scores using the 12-item OHS questionnaire and patients (not) experiencing a clinically relevant improvement for participating hospitals (*n* = 583)

Hospitals	n	Preoperative score	Postoperative score at 6 months	Change score	Patients with a clinically relevant improvement*	Patients with no clinically relevant improvement^#^
		[Mean ± SD]	[Mean ± SD]	[Mean ± SD]	[N (%)]	[N (%)]
Hospital 1	55	21.92 ± 7.31	39.71 ± 9.98	17.79 ± 9.18	45 (81.8%)	10 (18.2%)
Hospital 2	69	20.41 ± 7.04	40.68 ± 9.33	20.27 ± 10.73	59 (85.5%)	10 (14.5%)
Hospital 3	21	19.40 ± 6.04	38.35 ± 8.00	18.95 ± 7.70	19 (90.5%)	2 (9.5%)
Hospital 4	39	20.91 ± 8.14	37.64 ± 10.04	16.72 ± 11.66	30 (76.9%)	9 (23.1%)
Hospital 5	70	21.42 ± 7.39	41.18 ± 6.55	19.77 ± 9.37	62 (88.6%)	8 (11.4%)
Hospital 6	67	22.64 ± 7.86	40.92 ± 7.31	18.28 ± 9.88	56 (83.6%)	11 (16.4%)
Hospital 7	65	21.35 ± 7.51	42.72 ± 6.05	21.38 ± 8.06	62 (95.4%)	3 (4.6%)
Hospital 8	70	23.06 ± 7.60	42.61 ± 6.83	19.55 ± 8.77	64 (91.4%)	6 (8.6%)
Hospital 9	69	22.69 ± 8.31	41.15 ± 8.00	18.47 ± 9.67	57 (82.6%)	12 (17.4%)
Hospital 10	58	20.33 ± 7.98	39.15 ± 8.54	18.82 ± 9.83	49 (84.5%)	9 (15.5%)
*Overall*	*583*	*21.61* ± *7.63*	*40.75* ± *8.10*	*19.14* ± *9.58*	*503 (86.3%)*	*80 (13.7%)*

^*^ Minimal Important Change (MIC): OHS change ≥ 9.00 points; ^#^ OHS change < 9.00 points

Table 3 presents a more in-depth analysis of the characteristics of those patients who experienced a clinically relevant improvement (86.3%) and of those patients with an OHS change of less than 9 points (13.7%). Regarding the latter, those patients had higher preoperative OHS scores (27.74 vs. 20.64; *p* < 0.001), were more likely to take pain relief (*p* = 0.004) and to suffer from high blood pressure as a pre-existing condition (61.3% vs. 47.4%; *p* = 0.024). Differences regarding other characteristics (e.g., age, education, health status) could not be shown to be statistically significant.

**Table 3:** Overview of patients (not) experiencing a clinically important improvement (Minimal Important Change, MIC)

	OHS change < 9.00 (*n* = 80; 13.7%)	OHS change ≥ 9.00 (*n* = 503; 86.3%)	
	Mean (SD) or *n* (%)	Mean (SD) or n (%)	*P*
Age			
Mean (SD)	67.09 (9.97)	65.29 (9.88)	.144^3^
Gender			
Male	47.5%	36.4%	.057^4^
Female	52.5%	63.6%	
Preoperative OHS			
Mean (SD)	27.74 (8.71)	20.64 (6.98)	< .001^2^
Preoperative Body-Mass-Index (BMI)			
Mean (SD)	27.91 (5.00)	28.93 (5.23)	.107^1^
Educational attainment			
Secondary general school or less	31 (39.2%)	214 (43.0%)	.329^4^
Intermediate secondary school	35 (44.3%)	179 (35.9%)	
(Technical) University entrance qualification	13 (16.5%)	105 (21.1%)	
Health Status			
Good or better	48 (60.0%)	334 (66.5%)	.214^4^
Satisfactory	29 (36.3%)	137 (27.3%)	
Bad or worse	3 (3.8%)	31 (6.2%)	
Year of diagnosis			
2 years and less ago	38 (50.7%)	245 (50.7%)	.840^4^
3–5 years ago	15 (20.0%)	96 (19.9%)	
6–10 years ago	13 (17.3%)	69 (14.3%)	
11 years ago or more	9 (12.0%)	73 (15.1%)	
Intake of pain reliever			
Daily	18 (22.5%)	216 (43.1%)	.004^4^
4–6 times a week	7 (8.8%)	55 (11.0%)	
2–3 times a week	20 (25.0%)	77 (15.4%)	
1 time a week	8 (10.0%)	38 (7.6%)	
Less often or never	27 (33.8%)	115 (23.0%)	
Pre-existing conditions (the ten most frequently mentioned)			
Osteoarthritis	62 (78.5%)	388 (80.8%)	.625^4^
Back pain	46 (59.0%)	269 (55.8%)	.601^4^
High blood pressure	46 (61.3%)	236 (47.4%)	.024^4^
Gastrointestinal problems	14 (17.9%)	53 (11.1%)	.084^4^
Heart problems	11 (14.3%)	55 (11.4%)	.468^4^
Diabetes/Blood sugar	9 (11.7%)	47 (9.8%)	.607^4^
Lung problems	10 (13.2%)	34 (7.1%)	.067^4^
Depression	4 (5.3%)	38 (7.9%)	.414^4^
Rheumatism	5 (6.5%)	30 (6.3%)	1.000^5^
Cancer	3 (3.9%)	25 (5.2%)	.784^5^

Please note: ^1^two-sample t-test, ^2^Welch-t-test, ^3^Wilcoxon rank sum test, ^4^χ^2-test, ^5^Fisher’s exact test

Please find in the following the number of missings (those were taken into account for statistical calculations): Educational attainment (*n* = 1/5), Health Status (*n* = 0/1), Year of diagnosis (*n* = 5/20), Intake of pain reliever (*n* = 0/2), Osteoarthritis (*n* = 1/23), Back pain (*n* = 2/21), High blood pressure (*n* = 5/5), Gastrointestinal problems (*n* = 2/25), Heart problems (*n* = 3/21), Diabetes/Blood sugar (*n* = 3/23), Lung problems (*n* = 4/22), Depression (*n* = 2/24), Rheumatism (*n* = 3/27), Cancer (*n* = 4/24)

Furthermore, Table 4 shows the results of the multifactorial ANCOVA model; here, the adjusted OHS change scores for patients of participating hospitals are presented in detail. The adjusted change score for the top-performing hospital (number 7; 17.99 OHS points) is compared with the change score of the remaining nine hospitals. As shown, we were able to detect differences in the adjusted OHS mean change score from preoperative to 6-months postoperative between the top-performing hospital 7 and two other hospitals. Expanding further, hospital 4 (13.41 OHS points) and hospital 10 (15.13 OHS points) had notably lower mean OHS change scores compared with hospital 7 (hospital 4: -4.57; 95% CI: -7.67 to -1.48; *p* = 0.004; hospital 10: -2.86; 95% CI: -5.63 to -0.09; *p* = 0.043). However, it should be noted that this variation was not clinically relevant since it was below the MID threshold of 5.22 OHS points (see above). [Please see also Supplemental Material 5 presenting a forest plot displaying the difference in adjusted OHS scores by hospital.] However, the result of the corresponding ANCOVA *F*-test, which represents the global effect of the variable, was not significant; the results must therefore be regarded with caution.

**Table 4:** Preoperative and postoperative adjusted scores for participating hospitals using the 12-item OHS questionnaire (*n* = 583)

		*n*	Adjusted change score [Mean]	Comparison to top-performing hospital		
				Mean difference/Increase*	95% CI	*p* value
Model constant			40.27		35.42; 45.12	< 0.001
Hospitals	Hospital 7	65	17.99			0.141
	Hospital 1	55	15.39	-2.60	-5.39; 0.19	0.068
	Hospital 2	69	16.23	-1.76	-4.44; 0.92	0.199
	Hospital 3	21	14.47	-3.51	-7.38; 0.36	0.075
	Hospital 4	39	13.41	-4.57	-7.67; -1.48	0.004
	Hospital 5	70	16.50	-1.49	-4.13; 1.15	0.267
	Hospital 6	67	16.72	-1.27	-3.99; 1.45	0.361
	Hospital 8	70	17.56	-0.42	-3.06; 2.22	0.753
	Hospital 9	69	16.31	-1.67	-4.30; 0.96	0.212
	Hospital 10	58	15.13	-2.86	-5.63; -0.09	0.043
Gender	Female	362	16.29	-		0.355
	Male	221	15.65	-0.63	-1.97; 0.71	0.355
Nationality	German	566	17.26	-		0.194
	Other	17	14.68	-2.58	-6.47; 1.31	0.194
Health Status	Good or better	382	17.55	-		0.231
	Satisfactory	166	16.03	-1.52	-3.00; -0.04	0.044
	Bad or worse	34	17.17	-0.38	-3.25; 2.50	0.797
	Missing	1	13.13	-4.42	-20.13; 11.30	0.581
Duration of symptoms	1 year and less ago	119	17.46			0.364
	1–2 years ago	166	16.34	-1.12	-2.96; 0.72	0.231
	3–5 years ago	154	16.42	-1.04	-2.92; 0.84	0.277
	6–10 years ago	56	14.31	-3.15	-5.66; -0.64	0.014
	11 years ago or more	65	16.67	-0.79	-3.17; 1.58	0.513
	n.a	16	15.63	-1.83	-6.03; 2.37	0.393
	Missing	7	14.96	-2.50	-8.43; 3.43	0.409
Intake of pain reliever	Daily	234	13.80			0.191
	4–6 times a week	62	14.06	0.25	-1.99; 2.50	0.824
	2–3 times a week	97	13.70	-0.10	-2.04; 1.84	0.917
	1 time a week	46	14.58	0.78	-1.80; 3.35	0.553
	Less often or never	142	15.52	1.72	-0.10; 3.53	0.063
	Missing	2	24.17	10.37	-0.88; 21.61	0.071
Hip replacement other side	No	404	16.89			0.175
	Yes	122	15.73	-1.16	-2.76; 0.43	0.152
	Missing	57	15.29	-1.60	-3.81; 0.60	0.154
Pre-existing condition—Anemia or other blood disorders	No	543	13.49			0.023
	Yes	12	17.04	3.55	-1.01; 8.12	0.127
	Missing	28	17.39	3.90	0.64; 7.17	0.019
Pre-existing condition—High blood pressure	No	291	20.01			0.000
	Yes	282	19.11	-0.90	-2.25; 0.45	0.191
	Missing	10	8.79	-11.22	-16.58; -5.86	< .0001
Preoperative BMI		583		-0.03*	-0.16; 0.10	0.623
Preoperative OHS score		583		-0.76*	-0.86; -0.66	< 0.001

Furthermore, we were able to detect differences regarding certain patient characteristics (also Table 4). For example, patients with a satisfactory health status had significantly lower mean OHS change scores than patients with a good or better health status (-1.52, 95% CI: -3.00 to -0.04; *p* = 0.044). Also, patients with a duration of symptoms of between 6 and 10 years had significantly lower mean OHS change scores than those patients with a symptom duration of 1 year or less (-3.15, 95% CI: -5.66; -0.64; *p* = 0.014). Again, the global *p*-value was not significant for these factors either. Finally, patients with lower preoperative OHS scores had significantly higher mean OHS change scores that those with higher preoperative OHS scores (-0.76, 95% CI: -0.86 to -0.66; *p* < 0.001).

## Discussion

The objective of the present study was to detect hospital-related variations for THR between ten high-volume hospitals in Germany using the Oxford Hip Score (OHS) as a Patient-Reported-Outcome Measure (PROM). To the best of our knowledge, this is the first study to use an OHS change to calculate variations in THR when comparing hospitals in Germany. It is also the first study to use OHS change scores as a PROM outcome measure to calculate hospital-related variations for THR outside of the United Kingdom.

First, our study sample appears to be similar to representative THR patient cohorts from Germany [2] and England [26]. For example, our study population had a mean age of 65.54 years and was thus slightly younger than both the German patient group from 2020 (68.71 years) [2] and patients in England (approx. 68 years) [4]. Thereby, the percentage of patients between 80 and 89 years was smaller in our study (8.75%) compared with all German patients (17.38%) [2] and those from England (approx. 15%) [26]. The percentage of female patients in our study (62.1%) is very similar to the German national gender distribution; here, the percentage of female patients was reported to be 58.4% [2]. Furthermore, almost every second patient in our study (i.e., 48%) reported suffering from high blood pressure, which is slightly higher than that reported in England (40%) [4].

Second, regarding hospital-related variations for THR in Germany, we showed that patients receiving treatment in two specific hospitals had lower mean OHS change scores than patients receiving treatment in the top-performing hospital. However, the OHS change scores of both hospitals could not be shown to be clinically relevant since they were below the MID threshold of 5.22 OHS points [15]. Following this, we did not find any hospital-related clinically relevant variation for THR across ten high-volume hospitals in Germany. Nevertheless, this result should not be generalized without a more in-depth reflection. For example, we could see that the improvement of OHS points in the top-performing hospital could be shown to be clinically relevant for 95.4% of all patients (62 out of 65 patients). In contrast, in the least-performing hospital, only three out of four patients (30/39; 76.9%) experienced a clinically relevant improvement. One reason for this difference might be found in the patient characteristics, what however, does not seem to be plausible in our study. For example, patients treated in the top-performing hospital could be shown to be older, with slightly higher preoperative OHS values and patients also report a longer patient history compared with patients from the least-performing hospital. Another reason might be found in the underlying group MID threshold of 5.22 points [15]. This value was calculated based on a data set of the NHS patient-reported outcome measures that included 82,415 THR patients from England and Wales who underwent surgery between 2009 and 2011. It is possible that an analysis of more up-to-date data collected by surveying a representative patient sample from Germany might lead to other threshold values. However, so far, we are not aware of any published German evidence in this regard. For example, the above-mentioned systematic review of calculated MID values and their respective ranges for the OHS summarized evidence from the UK (5), Singapore (3), France (1) and the Netherlands (1).

Third, as mentioned above, patients would receive high-quality care in any of the assessed ten hospitals based on our PRO-based findings. However, this conclusion should also be viewed in relation to results from other quality assurance systems in Germany. For example, the German federal state quality assurance system [2] focuses on clinical areas and indicators with the potential for quality improvement and the intention to make the quality of care comparable across hospitals. In total, this quality assurance system consists of eight risk-adjusted performance measures for elective THR. Thereby, hospitals with measures below certain thresholds are required to justify these variances in a formal statement. If the justification is considered to be insufficient, a quality deficit will be assigned. The quality indicators refer to in-hospital consequences before and after surgery (e.g., complication rate, mobility at hospital discharge, confirmed diagnosis rate, prevention of falls measures), regardless of whether a long-term perspective has an impact on the quality results [27]. Regarding the participating hospitals in our study, nine of the ten hospitals did not have any quality deficits and, thus, reached a performance of 100%; both of the low-performing hospitals (i.e., with lower PRO results) were within this top-performing group.

Furthermore, the quality assurance systems from the Allgemeine Ortskrankenkasse (AOK) is based on claims data for those who are insured with the statutory health insurance AOK (approximately 24 Mio.). Here, the quality measures focus on the “long-term quality of hospitals for selected diseases or procedures and are based on measures of complications and adverse events. For example, two indicators analyze unplanned readmissions and surgical complications within the first year after THR” [27]. Regarding those results, only one hospital was labeled as a top-performer, six hospitals were middle-performers and three hospitals as low-performers. Again, we could not find a strong association between those results and our PRO-based findings. For example, the three hospitals with the largest PRO changes were labeled as middle performers and the only top performer was in sixth place in terms of OHS gains. In addition, we showed that eight hospitals meet the requirements of the EndoCert certification system and were certified either as an EndoProstheticsCenter (EPZ) or an EndoProstheticsCenter of Maximum Care (EPZmax). The certificates are issued by the EndoCert Limited, which is a subsidiary of the German Society for Orthopedics and Orthopedic Surgery [28]. The certification process requires to meet comprehensive quality targets regarding structure, processes, and outcomes. For example, an EPZ needs to treat at least 100 patients per year, an EPZMax at least 200 patients per year [29]. However, we did not find a clear association between EndoProstheticsCenter certifications and differences in the OHS change score. For example, two hospitals were not certified as an EndoProstheticsCenter in our study; while one hospital indeed showed statistically significant lower OHS change scores than the top-performing hospital, the second hospital was labeled as an EndoProstheticsCenter of Maximum Care (EPZmax).

Fourth, the mean observed (i.e., unadjusted) OHS change score of all ten hospitals was determined to be 19.14 ± 9.58 OHS points; thereby, it ranged between 21.38 ± 8.06 OHS points for the top-performing hospital and 16.72 ± 11.66 OHS points for the lowest-performing hospital. This result seems to be similar to those from the United Kingdom. For example, an analysis of the OHS change of 226,796 planned primary hip replacements between April 2008 and December 2016 based on UK National Joint Registry data [30] showed a positive change in self-reported OHS change scores from 17.7 points (95% CI: 16.4 to 19.0) in April 2008 to 22.9 points (95% CI: 21.8 to 23.9) in December 2016 [30]. In another study, Garriga and colleagues calculated the OHS change score for a total of 173,107 patients who underwent THR surgery, nested in 207 health areas (i.e., Clinical Commission Groups, CCGs) in England [30]. Here, the observed (i.e., unadjusted) mean OHS change by CCG ranged from 17.5 to 24.9 points. The authors then grouped the CCGs into five performance groups based on the estimated OHS change scores. Here, 23 of the CCGs were assigned to the lowest performance group with an OHS chance score of between 18.7 and 20.1 points, while another 23 CCGs were assigned to the top-performing group with an OHS change score of between 22.7 and 24.6 points. Thus, the difference between hospitals for the lowest and highest of five categories ranged between 2.6 and 5.9 OHS change points. In this study, models also indicated an association of higher surgical volume by surgeon and by hospital as well as private hospitals with better patient outcomes. This finding might be explained – at least to a certain amount – by the changing case mix of public hospitals (i.e., the percentage of treated complex patients). Furthermore, Garriga and colleagues also demonstrated an association of the proportion of less experienced physicians with poorer outcomes [20]. This seems comparable to our observed OHS change score differences between top-performing and lower-performing hospitals; here, the differences ranged between 2.86 and 4.57 OHS points, respectively. When comparing our findings to those from the two mentioned studies from the United Kingdom, one might assume similar results in the OHS change at first sight. Nevertheless, we have to consider the fact that our analysis focuses on OHS change scores across ten high-volume hospitals in Germany. As evidence has shown a positive volume-outcome relationship for THR surgery (e.g., [21, 30]), we might assume at least slightly lower mean OHS change scores in Germany when also including middle- or low-volume hospitals.

Finally, we could show that 13,7% of all patients in our study (80 of 583) did not experience a clinically relevant improvement. Thereby, we could see differences between the participating hospitals. For example, only 4,6% of all patients of the top performing hospital did not achieve a clinically relevant improvement. In contrast, 23,1% and 15,5% of all patients experienced a clinically relevant improvement regarding the two hospitals with statistically lower OHS change scores. In addition, we could see differences regarding certain patient characteristics; for example, patients without clinically relevant improvements were shown to have significant higher preoperative OHS scores (27.74) than patients with a clinically important improvement (20.64). A lower self-reported OHS score before THR surgery was significantly associated with higher OHS change scores. This finding is in line with international evidence, since studies from the UK and New Zealand have also demonstrated similar results [31–35]. In addition, patients without clinically relevant improvements reported significantly lower pain reliever usage; here, only 22,5% reported a daily intake compared with 41,1% regarding those patients who experienced a clinically relevant improvement. Thus, a lower self-reported usage of pain reliever was associated with lower OHS change scores; this finding is in line with a study from the United States [36].

Our findings should be considered in light of some limitations. We only included hospitals in the German Federal State Lower Saxony, where about 10% of all German hospitals are located [37]. All PROMs have possible ceiling effects, even though Lim and colleagues showed that the OHS did not exhibit a ceiling or floor effect, suggesting that the OHS should continue to be used as a valid measure of patient-reported outcomes for patients [38]. In our study, the postoperative OHS measurement was taken 6 months after surgery, as was the case in the above-mentioned studies of Garriga and colleagues [21, 30], Varagunam and colleagues [4] and NHS Digital [20]. However, some studies used a postoperative OHS measurement after 12 months. For example, Browne and colleagues showed that the twelve-month change scores reported were at least 2.1 points higher than the six-month change observed in the English PROMs program [39]. Furthermore, OHS-related PROMs may have some limitations as they do not collect data on high levels of physical activity [38, 40]. There might be several items that current patients consider to be relevant which are not included in the OHS, such as riding a bike or the ability to lower oneself onto and get up from the floor [41]. In addition, we tried to standardize pre-operative data collection across all participating hospitals as much as possible. In most cases, the postoperative survey was conducted in the clinic on the day of the surgery or the day prior to that. However, there were instances where the survey may have carried out during the consultation with the physician or anesthesiologist. In those cases, it took place a few days earlier, but not more than a week before the surgery. Therefore, we assume that the information provided is largely comparable, although an exact day-specific standardization across all participants was not conducted. We also did not specifically exclude patients awaiting bilateral hip replacement surgery. Regarding this, we did not ask whether a bilateral surgery was performed, nor did we define this as an exclusion criterion. Following this, some patients may have received bilateral hip replacement surgery. Another limitation refers to the fact that we did not collect how many surgeons were performing the hip replacement surgeries in each hospital nor how many procedures each of them performed individually. Both pieces of information would have been interesting to examine the impact of the performing physician on the outcome more closely. Finally, our study does not allow for an analysis of the development of the quality of care for THR surgery in Germany since we did not compare results over a longer period of time [30].

In sum, this study adds to the literature by detecting variations in the quality of care for ten high-volume hospitals in Germany for THR surgery based on patient-reported outcomes data from 583 patients. Following our study findings, we did not find any hospital-related clinically relevant variation for THR across the ten high-volume hospitals included. Indeed, we did find differences between the top-performing hospital and two hospitals; however, those differences were not clinically relevant since they were below the minimal important difference (MID) threshold. Based on this, we cannot show that patients would receive different levels of quality in any of the ten hospitals assessed. Furthermore, we could confirm international findings showing that both higher preoperative OHS scores and lower rates of pain relief usage was associated with lower change scores. We recommend studies to further explore hospital-related clinically relevant variation for THR which also include low-volume hospitals. Additionally, we recommend an evaluation of both MIC and MID thresholds in Germany.

## Supplementary Information

Below is the link to the electronic supplementary material.

Supplementary file1 (DOCX 189 KB)

## Data Availability

Data available on request from the corresponding author (martin.emmert@uni-bayreuth.de).
