# Supplementary material for: Variation in patient-reported outcomes after total hip replacement across ten high-volume hospitals in Germany: results from a multicenter, prospective, longitudinal Cohort Study
Source: Eur J Health Econ. 2025 Oct 29;27(3):757–68. doi: 10.1007/s10198-025-01858-4 (PMC13190762; doi:10.1007/s10198-025-01858-4)
Supplement: Supplementary file 1 — Supplementary file1 (DOCX 189 KB) [file 10198_2025_1858_MOESM1_ESM.docx]

Variation in Patient-Reported Outcomes after Total Hip Replacement Across Ten High-volume Hospitals in Germany: Results from a Multicenter, Prospective, Longitudinal Cohort Study

*Supplemental Material*

*Supplemental Material 1.* Key Characteristics of participating hospitals (n = 10)

|  | Hospital volume THR per annum quintil* | Certified endoprosthesis center** | Quality results of the federal state quality assurance system [2] | Result of AOK QSR quality assurance | Ownership[31] |
| --- | --- | --- | --- | --- | --- |
| Hospital 1 | High | Yes | 100.0% | Middle performer | Non-profit |
| Hospital 2 | Very High | No | 100.0% | Top performer | Private |
| Hospital 3 | Very High | Max | 100.0% | Low performer | Non-profit |
| Hospital 4 | High | No | 100.0% | Middle performer | Public |
| Hospital 5 | High | Yes | 87.5% | Middle performer | Non-profit |
| Hospital 6 | Very High | Max | 100.0% | Middle performer | Non-profit |
| Hospital 7 | Very High | Max | 100.0% | Middle performer | Non-profit |
| Hospital 8 | High | Yes | 100.0% | Middle performer | Non-profit |
| Hospital 9 | High | Max | 100.0% | Low performer | Non-profit |
| Hospital 10 | High | Max | 100.0% | Low performer | Public |
| * see methods section  ** Max: “EndoProthetikZentrum der Maximalversorgung” with at least 200 patients per year, yes: “EndoProthetikZentrum” with at least 100 patients per year | | | | | |

## *Supplemental Material 2.* Flow chart diagram of patient selection with THR surgery


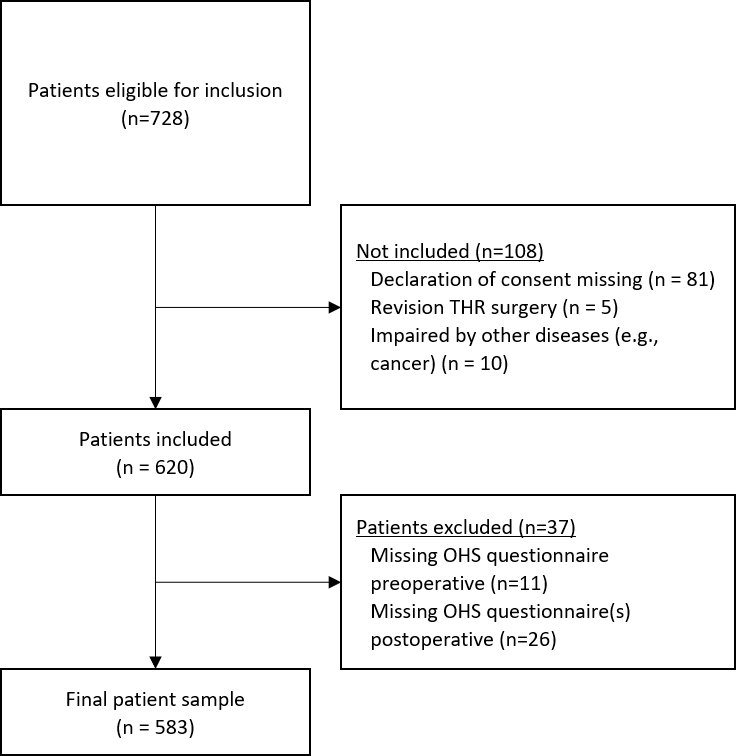


## *Supplemental Material 3.* Preoperative and postoperative unadjusted scores obtained using the 12-item OHS questionnaire (n = 583)

| During the past 4 weeks… | | Preoperative score | Postoperative score at 6 months | Change score | *p*-value |  |
| --- | --- | --- | --- | --- | --- | --- |
| Item | Content* | [Mean ± SD] | [Mean ± SD] | [Mean ± SD] |  |  |
| 1 | Usual level of hip pain | 0.48 ± 0.64 | 2.86 ± 1.27 | 2.38 ± 1.36 | <0.001 |  |
| 2 | Trouble with washing and drying | 2.57 ± 0.93 | 3.61 ± 0.70 | 1.04 ± 1.04 | <0.001 |  |
| 3 | Trouble with transport | 2.05 ± 0.80 | 3.48 ± 0.77 | 1.43 ± 0.96 | <0.001 |  |
| 4 | Putting on socks/stockings/tights | 1.93 ± 1.04 | 3.27 ± 0.94 | 1.34 ± 1.14 | <0.001 |  |
| 5 | Doing household shopping alone | 2.55 ± 1.06 | 3.60 ± 0.80 | 1.05 ± 1.08 | <0.001 |  |
| 6 | Walking time before severe pain | 2.43 ± 1.04 | 3.48 ± 0.92 | 1.05 ± 1.25 | <0.001 |  |
| 7 | Difficulty going up stairs | 2.06 ± 0.91 | 3.43 ± 0.80 | 1.36 ± 1.02 | <0.001 |  |
| 8 | Pain on standing up from sitting | 2.03 ± 0.90 | 3.51 ± 0.73 | 1.48 ± 1.04 | <0.001 |  |
| 9 | Limping when walking | 1.16 ± 1.02 | 3.23 ± 1.03 | 2.06 ± 1.41 | <0.001 |  |
| 10 | Sudden, severe pain from hip | 1.49 ± 1.19 | 3.52 ± 0.88 | 2.03 ± 1.38 | <0.001 |  |
| 11 | Work interference due to pain | 1.45 ± 0.85 | 3.33 ± 0.88 | 1.88 ± 1.08 | <0.001 |  |
| 12 | Pain in bed at night | 1.40 ± 1.13 | 3.44 ± 0.93 | 2.04 ± 1.32 | <0.001 |  |
| *Overall* | | *21.61 ± 7.63* | *40.75 ± 8.10* | *19.14 ± 9.58* | *<0.001* |  |
| * Each item is scored from 0 to 4, from most to least difficulty or severity. The scores are then added to produce a single figure with a range from 0 (most difficulties) to 48 (least difficulties).  All *p*-values were calculated using t-test (one sample). | | | | | | |

## *Supplemental Material 4.* Ridgeline plot displaying the distribution of the OHS difference by hospital (n = 583)


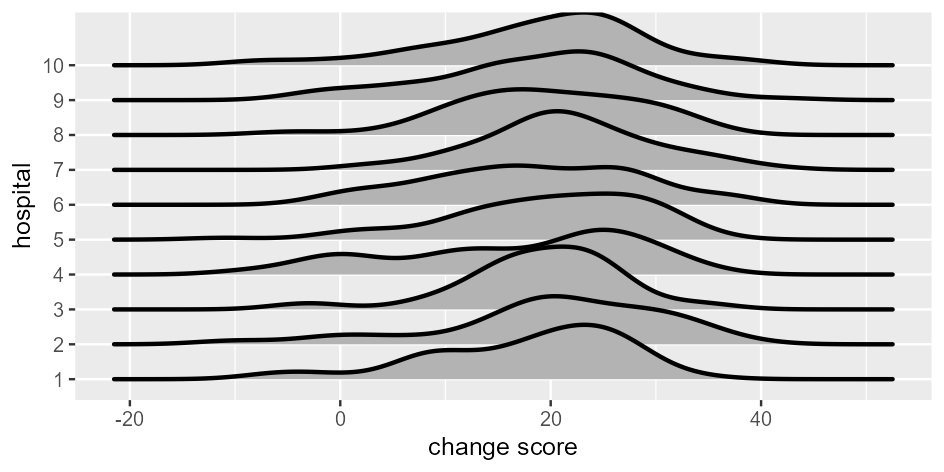


## *Supplemental Material 5.* Forest plot displaying the difference in adjusted OHS scores by hospital (n = 583)


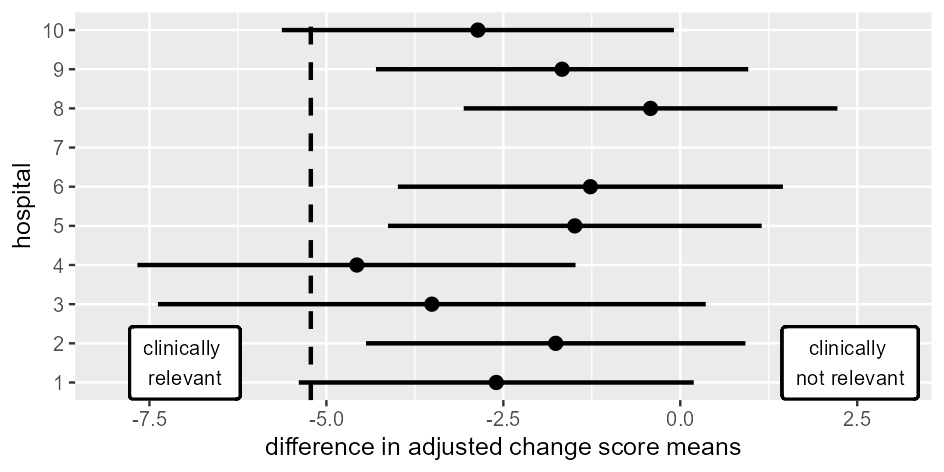


*Supplemental Material 6.* Overview of missing values for relevant variables from Table 1.

|  | Overall sample (n = 583) | | Hospitals | | | | | | | | | |
| --- | --- | --- | --- | --- | --- | --- | --- | --- | --- | --- | --- | --- |
|  | Mean (SD) or n | Range  or % | 1  (n=55) | 2  (n=69) | 3  (n=21) | 4  (n=39) | 5  (n=70) | 6  (n=67) | 7  (n=65) | 8  (n=70) | 9  (n=69) | 10  (n=58) |
| Preoperative Body-Mass-Index (BMI) |  |  |  |  |  |  |  |  |  |  |  |  |
| Mean (SD) | 28.78 (5.20) | 16.02-54.29 | 29.62 (6.04) | 29.55 (5.68) | 30.44 (5.72) | 29.08 (4.93) | 28.41 (5.06) | 29.21 (4.39) | 27.64 (4.22) | 29.04 (5.21) | 28.19 (5.07) | 27.91 (5.77) |
| Missings | 2 |  | 0 | 0 | 0 | 0 | 0 | 0 | 1 | 1 | 0 | 0 |
| Educational attainment |  |  |  |  |  |  |  |  |  |  |  |  |
| Secondary general school or less | 245 | 42.5 | 20 (37.0%) | 26 (38.8%) | 11 (55.0%) | 16 (41.0%) | 21 (30.4%) | 40 (59.7%) | 28 (43.1%) | 25 (36.2%) | 26 (37.7%) | 32 (56.1%) |
| Intermediate secondary school | 214 | 37.1 | 19 (35.2%) | 32 (47.8%) | 7 (35.0%) | 17 (43.6%) | 28 (40.6%) | 17 (25.4%) | 23 (35.4%) | 25 (36.2%) | 29 (42.0%) | 17 (29.8%) |
| (Technical) University entrance qualification | 118 | 20.5 | 15 (27.8%) | 9 (13.4%) | 2 (10.0%) | 6 (15.4%) | 21 (29.0%) | 10 (14.9%) | 14 (21.5%) | 19 (27.5%) | 14 (20.3%) | 8 (14.0%) |
| Missings | 6 |  | 1 | 2 | 1 | 0 | 0 | 0 | 0 | 1 | 0 | 1 |
| Health Status |  |  |  |  |  |  |  |  |  |  |  |  |
| Good or better | 382 | 65.6 | 41 (74.5%) | 42 (60.9%) | 10 (47.6%) | 25 (64.1%) | 53 (75.7%) | 42 (63.6%) | 45 (69.2%) | 47 (67.1%) | 44 (63.8%) | 33 (56.9%) |
| Satisfactory | 166 | 28.5 | 11 (20.0%) | 25 (36.2%) | 6 (28.6%) | 12 (30.8%) | 12 (17.1%) | 23 (34.8%) | 16 (24.6%) | 20 (28.6%) | 19 (27.5%) | 22 (37.9%) |
| Bad or worse | 34 | 5.8 | 3 (5.5%) | 2 (2.9%) | 5 (23.8%) | 2 (5.1%) | 5 (7.1%) | 1 (1.5%) | 4 (6.2%) | 3 (4.3%) | 6 (8.7%) | 3 (5.2%) |
| Missings | 1 |  | 0 | 0 | 0 | 0 | 0 | 1 | 0 | 0 | 0 | 0 |
| Year of diagnosis |  |  |  |  |  |  |  |  |  |  |  |  |
| 2 years and less ago | 283 | 50.7 | 22 (41.5%) | 41 (61.2%) | 7 (35.0%) | 24 (63.2%) | 29 (43.9%) | 33 (52.4%) | 23 (38.3%) | 34 (50.0%) | 36 (54.5%) | 34 (59.6%) |
| 3-5 years ago | 111 | 19.9 | 13 (24.5%) | 9 (13.4%) | 5 (25.0%) | 4 (10.5%) | 9 (13.6%) | 12 (19.0%) | 20 (33.3%) | 22 (32.4%) | 7 (10.6%) | 10 (17.5%) |
| 6-10 years ago | 82 | 14.7 | 10 (18.9%) | 11 (16.4%) | 5 (25.0%) | 5 (13.2%) | 9 (13.6%) | 9 (14.3%) | 7 (11.7%) | 5 (7.4%) | 14 (21.2%) | 7 (12.3%) |
| 11 years ago or more | 82 | 14.7 | 8 (15.1%) | 6 (9.0%) | 3 (15.0%) | 5 (13.2%) | 19 (28.8%) | 9 (14.3%) | 10 (16.7%) | 7 (10.3%) | 9 (13.6%) | 6 (10.5%) |
| Missings | 25 |  | 2 | 2 | 1 | 1 | 4 | 4 | 5 | 2 | 3 | 1 |
| Intake of pain reliever |  |  |  |  |  |  |  |  |  |  |  |  |
| Daily | 234 | 40.3 | 22 (40.0%) | 25 (37.3%) | 8 (38.1%) | 18 (46.2%) | 30 (42.9%) | 20 (29.9%) | 30 (46.2%) | 23 (32.9%) | 26 (37.7%) | 32 (55.2%) |
| 4-6 times a week | 62 | 10.7 | 2 (3.6%) | 5 (7.5%) | 4 (19.0%) | 5 (12.8%) | 11 (15.7%) | 13 (19.4%) | 4 (6.2%) | 8 (11.4%) | 7 (10.1%) | 3 (5.2%) |
| 2-3 times a week | 97 | 16.7 | 8 (14.5%) | 19 (28.4%) | 3 (14.3%) | 3 (7.7%) | 12 (17.1%) | 9(13.4%) | 8 (12.3%) | 13 (18.6%) | 12 (17.4%) | 10 (17.2%) |
| 1 time a week | 46 | 7.9 | 7 (12.7%) | 5 (7.5%) | 2 (9.5%) | 2 (5.1%) | 3 (4.3%) | 10 (14.9%) | 3 (4.6%) | 9 (12.9%) | 4 (5.8%) | 1 (1.7%) |
| Less often or never | 142 | 24.4 | 16 (29.1%) | 13 (19.4%) | 4 (19.0%) | 11 (28.2%) | 14 (20.0%) | 15 (22.4%) | 20 (30.8%) | 17 (24.3%) | 20 (29.0%) | 12 (20.7%) |
| Missings | 2 |  | 0 | 2 | 0 | 0 | 0 | 0 | 0 | 0 | 0 | 0 |
| Pre-existing conditions |  |  |  |  |  |  |  |  |  |  |  |  |
| Osteoarthritis | 450 | 80.5 | 42 (79.2%) | 47 (74.6%) | 14 (73.7%) | 32 (86.5%) | 57 (82.6%) | 49 (76.6%) | 53 (84.1%) | 56 (82.4%) | 53 (79.1%) | 47 (83.9%) |
| Missings | 24 |  | 2 | 6 | 2 | 2 | 1 | 3 | 2 | 2 | 2 | 2 |
| Back pain | 315 | 56.3 | 32 (60.4%) | 38 (62.3%) | 11 (61.1%) | 25 (65.8%) | 38 (54.3%) | 32 (50.0%) | 35 (55.6%) | 37 (54.4%) | 30 (44.1%) | 37 (64.9%) |
| Missings | 23 |  | 2 | 8 | 3 | 1 | 0 | 3 | 2 | 2 | 1 | 1 |
| High blood pressure | 282 | 49.2 | 30 (55.6%) | 36 (52.9%) | 12 (60.0%) | 21 (55.3%) | 25 (35.7%) | 40 (60.6%) | 30 (46.2%) | 33 (47.8%) | 25 (37.3%) | 30 (53.6%) |
| Missings | 10 |  | 1 | 1 | 1 | 1 | 0 | 1 | 0 | 1 | 2 | 2 |
| Gastrointestinal problems | 67 | 12.1 | 5 (9.4%) | 6 (9.7%) | 4 (22.2%) | 7 (18.4%) | 6 (8.7%) | 3 (4.5%) | 8 (12.5%) | 10 (14.7%) | 9 (13.6%) | 9 (17.3%) |
| Missings | 27 |  | 2 | 7 | 3 | 1 | 1 | 1 | 1 | 2 | 3 | 6 |
| Heart problems | 66 | 11.8 | 4 (7.5%) | 7 (11.3%) | 3 (15.8%) | 5 (12.8%) | 6 (8.6%) | 4 (6.0%) | 8 (12.5%) | 10 (14.7%) | 10 (14.9%) | 9 (18.0%) |
| Missings | 24 |  | 2 | 7 | 2 | 0 | 0 | 0 | 1 | 2 | 2 | 8 |
| Diabetes/Blood sugar | 56 | 10.1 | 5 (9.4%) | 11 (17.2%) | 3 (16.7%) | 5 (13.2%) | 5 (7.1%) | 10 (15.4%) | 5 (7.9%) | 2 (2.9%) | 4 (6.1%) | 6 (11.5%) |
| Missings | 26 |  | 2 | 5 | 3 | 1 | 0 | 2 | 2 | 2 | 3 | 6 |
| Lung problems | 44 | 7.9 | 2 (3.8%) | 7 (11.1%) | 3 (15.8%) | 3 (7.9%) | 8 (11.6%) | 3 (4.5%) | 1 (1.6%) | 5 (7.4%) | 7 (10.6%) | 5 (9.8%) |
| Missings | 26 |  | 2 | 6 | 2 | 1 | 1 | 1 | 1 | 2 | 3 | 7 |
| Depression | 42 | 7.6 | 7 (13.2%) | 5 (8.1%) | 3 (17.6%) | 2 (5.3%) | 6 (8.6%) | 4 (6.2%) | 4 (6.3%) | 3 (4.4%) | 3 (4.5%) | 5 (9.4%) |
| Missings | 28 |  | 2 | 7 | 4 | 1 | 0 | 2 | 2 | 2 | 3 | 5 |
| Rheumatism | 35 | 6.3 | 0 (0.0%) | 2 (3.2%) | 2 (11.1%) | 3 (7.9%) | 4 (5.8%) | 8 (12.5%) | 4 (6.3%) | 3 (4.5%) | 4 (6.0%) | 5 (9.8%) |
| Missings | 30 |  | 2 | 7 | 3 | 1 | 1 | 3 | 1 | 3 | 2 | 7 |
| Cancer | 28 | 5.0 | 3 (5.7%) | 6 (9.4%) | 2 (10.5%) | 2 (5.4%) | 0 (0.0%) | 2 (3.1%) | 5 (7.8%) | 2 (2.9%) | 3 (4.5%) | 3 (5.9%) |
| Missings | 28 |  | 2 | 5 | 2 | 2 | 2 | 2 | 1 | 2 | 3 | 7 |
